# Variance components of ratings of physician-patient communication: A generalizability theory analysis

**DOI:** 10.1371/journal.pone.0252968

**Published:** 2021-06-10

**Authors:** Nicole Röttele, Christian Schlett, Mirjam Körner, Erik Farin-Glattacker, Andrea C. Schöpf-Lazzarino, Sebastian Voigt-Radloff, Markus A. Wirtz

**Affiliations:** 1 Institute of Medical Psychology and Medical Sociology, Faculty of Medicine, University of Freiburg, Freiburg, Germany; 2 Section of Health Care Research and Rehabilitation Research, Medical Center–University of Freiburg, Faculty of Medicine, University of Freiburg, Freiburg, Germany; 3 Institute for Evidence in Medicine (for Cochrane Germany Foundation), Faculty of Medicine and Medical Center, University of Freiburg, Freiburg, Germany; 4 Department of Research Methods, Freiburg University of Education, Freiburg, Germany; Murcia University, Spain, SPAIN

## Abstract

**Background:**

The ratings of physician-patient communication are an important indicator of the quality of health care delivery and provide guidance for many important decisions in the health care setting and in health research. But there is no gold standard to assess physician-patient communication. Thus, depending on the specific measurement condition, multiple sources of variance may contribute to the total score variance of ratings of physician-patient communication. This may systematically impair the validity of conclusions drawn from rating data.

**Objective:**

To examine the extent to which different measurement conditions and rater perspectives, respectively contribute to the variance of physician-patient communication ratings.

**Methods:**

Variance components of ratings of physician-patient communication gained from 32 general practitioners and 252 patients from 25 family practices in Germany were analyzed using generalizability theory. The communication dimensions “shared decision making”, “effective and open communication” and “satisfaction” were considered.

**Results:**

Physician-patient communication ratings most substantially reflect unique rater-perspective and communication dimension combinations (32.7% interaction effect). The ratings also represented unique physician and rater-perspective combinations (16.3% interaction effect). However, physicians’ communication behavior and the observed communication dimensions revealed only a low extent of score variance (1% physician effect; 3.7% communication dimension effect). Approximately half of the variance remained unexplained (46.2% three-way interaction, confounded with error).

**Conclusion:**

The ratings of physician-patient communication minimally reflect physician communication skills in general. Instead, these ratings exhibit primarily differences among physicians and patients in their tendency to perceive shared decision making and effective and open communication and to be satisfied with communication, regardless of the communication behavior of physicians. Rater training and assessing low inferential ratings of physician-patient communication dimensions should be considered when subjective aspects of rater perspectives are not of interest.

## Introduction

Ratings of physician-patient communication (PPC) provide guidance for many important decisions in health research, health care delivery and health care professional trainings. In the field of health research, these ratings serve to promote evidence-based medicine. In doing so, ratings of PPC are used to investigate the effects of PPC on relevant clinical outcomes (e.g., patient quality of life, satisfaction with care and health status) [1–3]. The ratings are applied to assess physicians’ communication skills, to reveal needs for improvement and to derive appropriate approaches for interventions [1, 2]. Finally, they are used to evaluate such interventions and to decide whether they are effective and worthy of further implementation [4, 5]. In regard to health care delivery, these ratings serve as quality criteria for health facilities [6, 7]. In terms of health care professional training programs, ratings of PPC are incorporated to evaluate the communication competence of students, to assess learning needs and to justify modifications of curricula [8]. Accordingly, a complete understanding of PPC ratings is vital to progress and to defensible decision making in research, education and health care delivery.

However, there is no gold standard, accepted measure across settings which is used to assess ratings of PPC. For example, the different measures of PPC vary in the dimensions of PPC they assess (e.g. relationship building, promoting information exchange, facilitating patient involvement or satisfaction with the medical encounter) [9, 10]. In addition, measures of PPC also differ in the perspectives from which the ratings are made (e.g., physicians, patients, neutral observers) [11]. Thus, depending on the measure used, the ratings reflect PCC under specific measurement conditions. However, usually the aim is not to evaluate the quality of PPC under specific measurement conditions but to draw generalizable conclusions across different situations and conditions. For example, a neutral observer may assess a physicians’ communication competence using the OPTION scale [12] to evaluate an intervention program aiming to improve PPC. Then, one may not be interested in physicians’ communication competence how it is perceived by this specific rater on the specific dimension the OPTION scale assesses. Instead, one may be interested in the physicians’ overall communication competence across all possible measurement conditions (e.g. across all communication dimensions and all possible raters). Thus, it is important to reveal to what degree the assessed ratings reflect the construct of interest itself rather than its specific operationalized variant. This can be done by investigating the amount of score variance of the assessed ratings attributable to the specific measurement conditions. The less score variance is attributable to a specific aspect of the measurement situation, the more generalizable are the assessed ratings over this specific aspect.

Due to the possible variations in measurement conditions when investigation PPC, there are multiple sources of variance, which may contribute to the total score variance of PPC ratings. First, score variance may be due to the observer perspective [13, 14]. Physicians and patients may have different consistent tendencies to rate PPC quality as either high or low, which is also known as leniency or severity bias [15]. This bias may be specific to particular communication dimensions or particular physicians, leading to interaction effects of rater perspective and communication dimensions or rater perspective and physicians [14]. For example, in the case of the dimension satisfaction with the medical encounter, there is evidence, that patients tend to be more satisfied than their physicians when individual encounters have been rated from both perspectives [16, 17]. Furthermore, in a meta-analysis of six studies investigating shared decision making (SDM) physician and patient ratings proved to be weakly associated, while physicians rated SDM to be higher [18] (r_polyc_ = .15; 95% CI [0.08–0.21]). Two additional studies, consulting neutral observers and patients also indicate that there may be score variance in PPC ratings due to the observed perspective: observers and patients did not agree in their ratings of the same consultation. Thus, the ratings may depend more on the perspective of the assessing person than on the quality of PPC itself [19, 20].

Second, there may be score variance of PPC ratings due to an interaction effect of the analyzed perspective and the assessed communication dimensions. Particularly, physicians’ or patients’ impressions created in one area may influence their judgements in another area (halo effect). This phenomenon will lead to correlations on the different communication dimensions among ratings from a particular perspective [13]. For example, patient satisfaction with the medical encounter may also lead to higher ratings of information exchange and SDM but may not increase physicians’ ratings. Third, the assessed communication dimensions as well as an interaction effect of communication dimensions and investigated physicians may explain the score variance of PPC ratings. For example, ratings on satisfaction exhibit ceiling effects [15, 16]. Hence, observing the communication dimension of satisfaction may indicate a higher quality of PPC than observing other dimensions of communication, such as information exchange or SDM.

Previous studies did not reveal which of these specific characteristics of the measurement situation contribute to the variance of PPC ratings. When investigating the accuracy of PPC ratings, these studies typically focus on one global measurement error [20–22]. In doing so, all variance components accountable to the investigated type of error are incorporated into one single error term. None of these studies examines variability due to physician characteristics, rater perspective, or investigated dimensions of PPC and potential interaction effects between these factors simultaneously.

Generalizability theory (g-theory) allows simultaneous examination of multiple sources of variance [23]. By applying a generalizability study (g-study), it can be revealed how much of the score variance of PPC ratings is due to physicians’ communication behavior, raters’ perspective, communication dimensions, and interactions. Thus, in the context of PPC, g-theory outperforms previous used methods to investigate generalizability of ratings as it allows to separate the variability of ratings scores in different variance components instead of incorporating them into one global, unresolved error term. This process allows decision makers to enhance their understanding of the information included in the ratings and to draw more valid conclusions concerning the generalizability of PPC ratings across different measurement conditions. Two previous studies applying g-theory in the context of PPC investigated students’ communication competence in the field of health care professional training programs [24, 25]. Both studies investigated PPC ratings assessed from observers. G-theory proved to be effective as it allowed to decompose variance components differentially. However, to our knowledge, there is no study applying g-theory to investigate PPC ratings assessed from self-report measures.

In the presented study we investigated variance components of PPC ratings gained from physicians’ and patients’ perspectives. To represent the multidimensional characteristic of PPC, variance components of PPC ratings were analyzed on three communication dimensions representing two major aspects of PPC and one global indicator of the quality of PPC using g-theory. The observed communication dimensions are SDM, effective and open communication and satisfaction with the overall communication.

## Methods

### Setting and study population

Cross-sectional survey data measuring the quality of PPC from 48 general practitioners (GPs) and 302 back pain patients belonging to 33 family practices were collected in Northern Bavaria (Germany). Data were assessed from September 2018 to August 2020 as part of the “Well Informed Physicians and Patients (GAP)” trial [26]. The trial was funded by the German Innovation Fund (Federal Joint Committee; Grant: 01NVF17010) and is registered in the German Clinical Trials Register (DRKS00014279). The study was approved by the ethics committee of the Albert-Ludwigs-University Freiburg (No. 559–17). All participants provided written informed consent to participate.

*The Institute of General Practice at the University Medical Center Erlangen* recruited GPs who recruited patients in their family practices. All GPs in Bavaria were eligible and were contacted in a two-step process: 1. Invitation by postal/fax through the Bavarian Association of General Practitioners (BHÄV). 2. By personal mail or telephone contact of the GPs. Participating GPs received information concerning the study, regulations for enrolling in the intervention program, and participation documents for the GP practice and the patients. Eligible patients received brief study information at their first consultation and were invited to participate in the study. If interest was confirmed, patients were informed and enrolled in the study by physicians. To facilitate recruitment, participating physicians received an incentive of 34€ per enrolled patient, and participating patients received a book voucher for 25€. Eligible patients were aged 18 years and older, were insured with a company health insurance fund and consulted the participating GP due to back pain symptoms. Sufficient German language skills to complete the study questionnaire were also required for study inclusion.

The intervention consisted of an online portal for the GPs and patients containing evidence-based information on treatment options for back pain. Once the GPs were recruited they were randomized into either control or intervention group. The allocation ratio was 2:1 for intervention and control group. GPs in the intervention group used the online portal during the consultations with their patients included in the study. Furthermore, they gave their patients login data for the online portal. Thus, their patients also had access to the online portal after the consultation. GPs in the control group continued to perform their usual consultation routines and their patients did not get access to the online portal.

Physician and patient ratings on the three dimensions measuring the quality of PPC were assessed using online and paper-pencil questionnaires. In particular, for physicians in rural areas and insufficient internet access as well as for patients completing questionnaires at the physician’s office, paper-pencil questionnaires were more practical. Physicians were invited to complete an online questionnaire directly after recruiting the last patient and to give an overall rating of the quality of PPC for all their consultations with their enrolled patients. Physicians who did not fill in the online questionnaires after a survey period of four weeks, were sent paper-pencil questionnaires. To fill in the paper-pencil questionnaires, physicians had an additional time of six weeks (to ensure enough time for postal mail). All patients completed the questionnaire directly after the consultation and evaluated the quality of the PPC of this single consultation using paper-pencil questionnaires.

### Patient and physician instruments

The three dimensions indicating the quality of PPC were assessed using two dyadic instruments (consisting of a physician and patient version), namely, the SDM questionnaire (SDM-Q) [27–29], the short version of the satisfaction scale of the P.A. INT questionnaire [30–32], and the effective and open communication scale of the KOVA questionnaire for patients [33]. The latter was adapted for physicians given that a physician version is not available. Additionally, instructions and items for the physician’s questionnaire were adapted to provide an overall rating for all consultations. To validate these adaptations, the physicians’ questionnaire was pretested in a sample of 7 GPs. All items of the questionnaires are presented in the S1 File.

The SDM-Q assesses SDM and consists of 9 items with participants responding on a 6-point Likert scale (ranging from “1” = “completely disagree” to “6” = “completely agree”; e.g. “My doctor asked me which treatment option I prefer” /” I asked my patients which treatment option they prefer”). A raw total score can be calculated by adding up the scores of all items. This score can be standardized into a scale ranging from 0 to 100 with zero indicating the lowest level of perceived SDM and 100 indicating the highest level of perceived SDM. The psychometric properties of the SDM-Q were evaluated in two previous studies in consultations with 324 patients with chronic disease and 29 GPs and other medical specialists [27, 29]. Both the patient version (α = .94) [27] and the physician version (α = .88) [29] showed good internal consistency. The patient version also revealed good construct validity with all items loading on one factor, which accounted for 62.4% of the total variance. Furthermore, a former version of the scale showed moderate correlation with the subdimensions of the Perceived Involvement in Care Scale [27].

The short version of the satisfaction scale of the P.A. INT questionnaire is a two-item scale assessing global satisfaction with PPC in medical consultations, with participants responding on a 5-point Likert scale (ranging from “1” = “completely disagree” to “5” = “completely agree”; e.g. “I am satisfied with the result of the consultation” / “I am satisfied with the result of the consultations”). Higher scores reflect higher satisfaction. A previous study with 474 inpatient rehabilitants and 60 physicians investigated the reliability of the questionnaire. High internal consistency was observed for the physician (α = .96) and patient versions (α = .95) of the satisfaction scale [32].

The scale effective and open communication of the KOVA questionnaire is a 10-item scale with participants responding on a 6-point Likert scale (ranging from “1” = “strongly disagree” to “6” = “strongly agree”; e.g. “Your physician listened carefully when you wanted to say something” / “I listened carefully when my patients wanted to say something”). A raw total score can be calculated by averaging the single scores of all 10 items. This raw total score can be standardized by transformation into a scale ranging from 0 to 100. Higher values indicate more open and effective communication behavior of the physician. The scale showed good internal consistency (α = .90) in a previous study with 703 back pain patients [33].

### Statistical methods

A missing data analysis was conducted. Surveys with greater than 30% scale items missing were excluded from analysis [34]. Missing data were imputed using the expectation–maximization algorithm [35, 36]. All scale scores were transformed from 0 to 100. Internal consistency of the patient and physicians’ versions of the three scales was assessed by Cronbach’s alpha coefficients. As patients were nested within physicians, the intraclass correlation coefficient (ICC) was calculated to investigate patient score variance attributable to physicians [37]. A higher value indicates high consistency among the patient ratings of the same physician, i.e., the variation of patient scores nested within the same physicians is relatively smaller than the variation between patient scores of different physicians. Three paired-samples t-tests were calculated to detect differences from physicians’ and patients’ mean scores of the examined communication dimensions. A significance level of p < .05 was chosen.

To identify the variance components representing the contribution of the investigated measurement conditions (facets) and their interactions to the total score variance of communication ratings, a g-study was conducted with a crossed two-facet random effects measurement design (P x R x C) [23, 38]. The object of measurement (differentiation facet) was physicians (P). Instrumentation facets included perspectives from which the scores were obtained (R) and communication dimensions for which the ratings were assessed (C). All facets were considered random. As physicians provide an overall rating for communication with all their patients, patient ratings belonging to the same physician were also summarized by calculating means. This process allowed for a crossed design. The g-study was performed with EduG 6e software [39], and all other statistical analyses were performed with SPSS version 26 [40].

## Results

A total of 252 patient questionnaires and 32 physician questionnaires from 25 family practices could be included in the analysis. Thirty-nine patient questionnaires and 16 physician questionnaires were excluded because there was no questionnaire from the second perspective. Another 11 patient questionnaires were excluded due to missing data. Table 1 shows the sociodemographic data of the included physicians and patients.

**Table 1:** Characteristics of patients and physicians included in the study.

	Patients		Physicians	
	Mean	*SD*	Mean	*SD*
Age (years)	47.01	14.29	49.63	9.42
Work experience (years)	N/A	N/A	15.27	10.23
working hours per week	N/A	N/A	43.66	13.61
	*N* (total = 252)	%	*N* (total = 32)	%
Gender				
Male	101	40.1	20	62.5
Female	150	59.5	10	31.3
Type of practice	N/A	N/A		
Joint practice			20	62.5
Group practice			2	6.3
Single-partner practice			7	21.9
Other			1	3.1
German mother language	240	95.2	N/A	N/A
Educational level			N/A	N/A
Low	71	28.2		
Medium	98	38.9		
High	72	28.6		

*Note*. N/A not applicable.

The range of patients nested within physicians was from 1 to 38 (M = 7.88; SD = 7.83). The ICCs of patients’ ratings were not significant for SDM and were only small for satisfaction and effective and open communication (ICC = .08). Thus, the same variability is noted in the communication scores from one patient to another patient of the same physician as from one patient of one physician to another patient of another physician.

**Fig 1:**
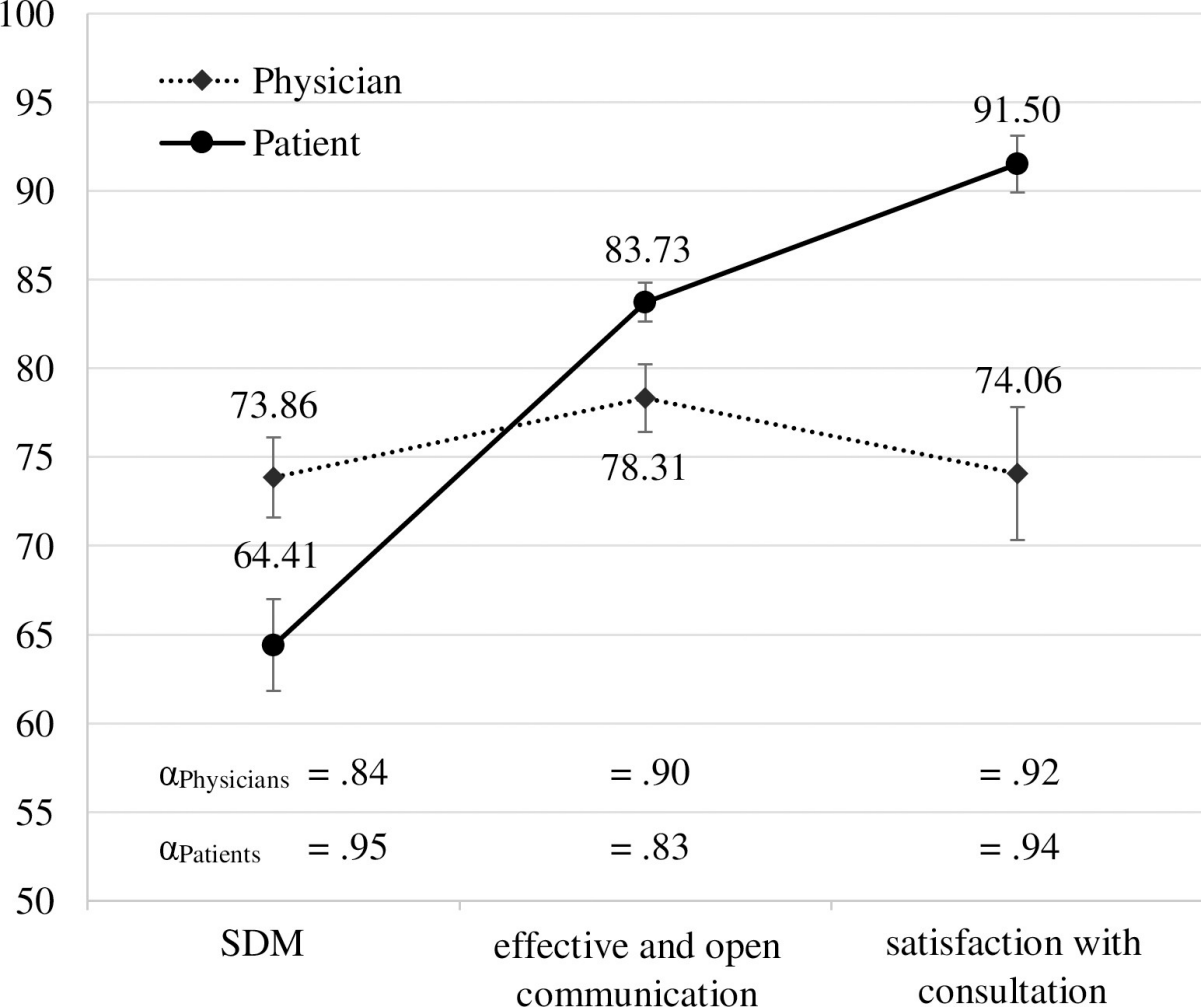
Mean scores, standard errors and Cronbach’s α for physician and patient rating scales.

Mean scores, standard errors and Cronbach’s α for the ratings from physicians and patients’ perspectives of the three communication dimensions are shown in Fig 1. T-test results indicate that physicians’ and patients’ rating levels differ significantly for all three dimensions, t(31) = 3.11, p = .004, d = 0.55 (SDM); t(31) = -2.65, p = .012, d = 0.47 (effective and open communication); t(31) = -4.10, p < .001, d = 0.72 (satisfaction). While physicians rate SDM to be higher, patients judge effective and open communication as well as satisfaction to be higher. All scales assessed revealed high internal consistency for both perspectives (α > .83).

Table 2 provides variance components and G-coefficients for the ratings of PPC. The variance component attributable to physician effects indicates to what extent individual physicians differ in overall PPC. This variance component accounts for 1% of the total score variance.

**Table 2:** G-study estimates of variance components and G-coefficients.

Effect	VC^a^	Contribution to variance (%)
P	2.791	1.0
R	-25.533	0.0
C	10.712	3.7
P x R	46.808	16.3
P x C	-5.498	0.0
R x C	93.752	32.7
P x R x C	132.337	46.2
G-coefficient relative		0.06
G-coefficient absolute		0.04

*Note*.

^a^ Estimated variance components. P = physician; R = rater-perspective; C = communication dimension; P x R = physician x rater-perspective; P x C = physician x communication dimension; R x C = rater-perspective x communication dimension; P x R x C = physician x rater-perspective x communication dimension.

**Fig 2:**
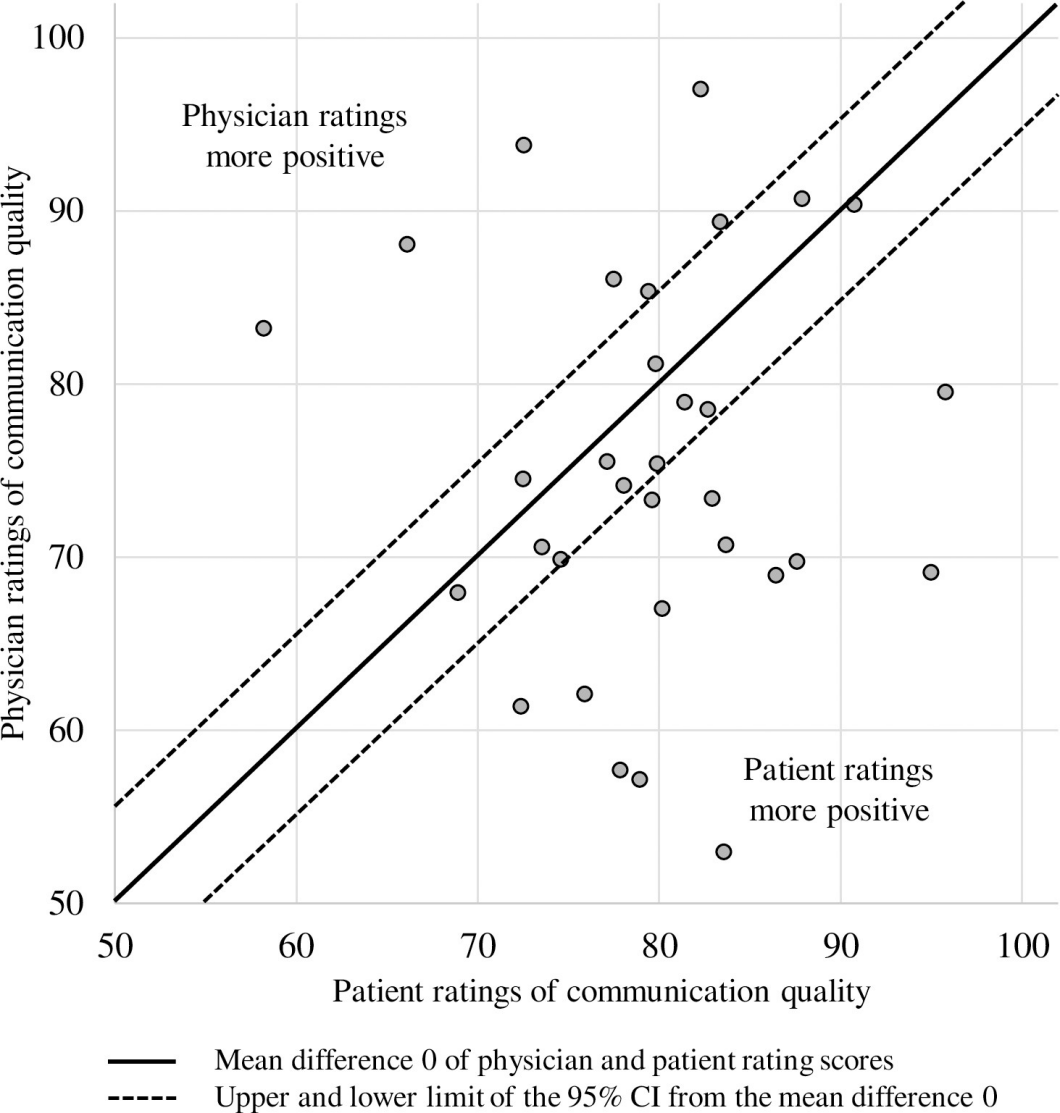
Interaction effect of perspective facet and physician facet.

The variance component for physician and perspective interaction (P x R) reflects variance attributable to rater perspectives and a particular physician. Depending on the particular physician, the congruence of rater perspectives varies systematically. This interaction accounts for approximately 16% of the total score variance. This interaction effect is shown in Fig 2. The difference in the overall PPC ratings between the two perspectives is for 12 physicians within the 95% CI with a mean difference of 0 indicating concordant ratings (diagonal). However, 13 physicians provided lower ratings for their own overall communication quality than their patients, whereas 7 physicians gave higher ratings.

The interaction effect of perspective and communication (R x C) becomes evident in Fig 1: leniency and severity biases of rater perspectives are communication dimension specific. The g-study further reveals that this interaction effect explains 33% of the total variance in scores. Variance due to communication dimension indicates to what extent ratings on a specific dimension of PPC differ from ratings of other dimensions of PPC and contributes 4% to score variance. The residual variance component (P x R x C) accounts for approximately 46% of the score variance. This component reflects variance attributable to the three-way interaction and is confounded with error variance. The low g-coefficients indicate that physician and patient ratings have limitations in reliably assessing the overall communication of individual physicians.

## Discussion

PPC ratings were minimally able to distinguish among the communication competence of individual physicians. Instead, the ratings more substantially reflect differences among physicians and patients in their tendency to perceive SDM, effective and open communication and to be satisfied with the communication. Thus, patients evaluate communication more effectively and openly and are more satisfied with communication in the consultation than their counterparts. In contrast, physicians perceive that they do better at SDM than their patients perceive. Furthermore, PPC ratings reflect physicians’ and patients’ leniency and severity bias unique to specific physicians. Thus, some physicians are rated in their communication more positively by their patients than indicated by their self-ratings. However, other physicians are rated more negative by their patients than indicated by their self-ratings. Finally, a high amount of score variance cannot be explained despite investigating the different variance facets P, R and C as well as their interactions.

The findings of this study are consistent with previous studies applying g-theory to examine ratings from neutral observers [24, 25] or in other contexts [14, 41] and are emphasized by studies investigating PPC ratings using other methods [18, 42]. Two studies examined ratings of PCC assessed from neutral observers to evaluate students communication competence in the context of health professional training programs [24, 25]. It was shown that students’ communication competence could only be explained to a small extent (3% [24]; 11% [25]). This was also shown by a study examining the cultural competency of attending physicians using g-theory [41]. Physicians’ cultural competency could only be explained to a small extent (3%). Thus, the physician effect was also very low. Instead, the ratings also reflected the observer’s unique perceptions of specific physicians (47%; interaction effect of observer and physician) [41]. A meta-analysis investigating a broad variety of ratings in the context of psychological research showed similar results [14]. Approximately 37% of the score variance was attributable to raters’ unique perceptions of particular targets and to raters’ different tendencies to interpret the investigated rating scales. The latter is further emphasized by studies examining interrater agreement of physician and patient ratings [18, 42]. They indicate that physicians and patients tend to perceive single communication dimensions differently, whereas physicians rated the presence of SDM higher [43].

The different tendencies of the rater perspectives to rate the single communication dimensions of PPC may be due to several reasons. Different attributions of the meanings of the communication dimensions could provide different ratings from the perspectives [14]. Since patients are less familiar with the language of the items and instructions, their ratings may reflect broader aspects of communication than those from physicians [44]. Especially in the case of SDM ratings, physicians and patients may refer to distinctive content meanings given that patients’ ratings do not only reflect the decision-making process. Instead, they may also reflect other aspects inherent in the patient’s perspective, i.e., the alternative they focus on, their assessment of constraints on choices, and their evaluation of how good the best possible solution is [45]. However, physicians may focus on their strengths and overlook their weaknesses when evaluating their own competence to involve patients in decision making. In doing so, they enhance their self-esteem and produce a self-serving bias. Furthermore, the importance of SDM is forced in health professionals’ training and in evaluations of health care delivery quality. This phenomenon may lead physicians to reveal higher SDM ratings due to social desirability [46].

However, other sources of disagreement may be relevant regarding ratings of satisfaction with communication in the consultation. As subjective perception may be more important than the evaluation of physician performance, patients may be more satisfied with communication in consultations than physicians due to different expectations concerning medical encounters [47, 48]. Although physicians are educated about what aspects constitute good PPC, they may have expectations concerning medical encounters other than their patients, who may have formed their expectations due to former experiences. Thus, physician satisfaction ratings may more reflect on the degree to which medical standards are met. Patient ratings, however, may reflect to what extent their expectations were met based on former experiences. Another reason for the higher satisfaction ratings of patients could be a social desirability response bias. They may feel that positive comments are more likely to be accepted by survey administrators and therefore report greater satisfaction than they actually feel. Furthermore, being at the family practice when completing the questionnaires may have evoked the desire of patients to integrate themselves with medical staff, leading to more favorable ratings [47]. Finally, a halo effect may produce higher satisfaction ratings from patients, resulting in the contribution of unique impressions to the ratings [14].

The consequences of the high amount of variance in PPC ratings due to rater-perspective interaction effects should be considered separately for every single communication dimension as they underlie different conceptual frameworks of communication [11]. For example, satisfaction ratings are considered to be subjective judgments of an interaction between two individuals. Therefore, the individual perception and experience of the two parties is of interest. Thus, both variance due to rater perspective and variance due to the interaction of rater perspective and physician may reflect valid information. However, regarding ratings of effective and open communication behavior of the physician, communication is considered an observational behavior of one individual. It is not of interest how the behavior was interpreted by the perspectives but how the physicians acted. Hence, variance due to rater perspectives would be a source of error. In the case of SDM, both the observational behavior of the interaction and the perceived involvement could be of interest [49]. Thus, whether rater-perspective variance is a problem in SDM ratings depends on the research question.

To further facilitate the interpretation of PPC ratings, further research is needed. The development and evaluation of methods aiming to improve the reflection of study participants when responding to items of PPC may reduce rater biases, such as self-serving bias and halo effects. Additionally, investigating the decision-making process underlying when giving ratings of PPC would be valuable. Further examination of which characteristics lead to different ratings of the two perspectives for particular physicians will be performed. Furthermore, investigating further variance components that could not be explained in this study may provide a deeper understanding of the ratings. This information may also reveal additional indicators for interventions aiming to improve PPC. Additionally, conducting a g-study may be instructive taking into account not only self-assessments by physicians and patients, but also assessments by neutral observers. Finally, further research is necessary that goes beyond samples in the primary care setting with the indication of back pain.

### Limitations

When interpreting the results, several limitations should be mentioned. First, physicians provided an overall rating of all patients included in the study. A study design where physicians give ratings for every single consultation instead of one overall rating may further have explained more score variance. However, as providing single ratings is an immensely greater effort for physicians, gathering overall ratings is a realistic scenario for studies observing physicians’ ratings. Additionally, the overall rating of physicians may have produced systematic observer bias. For example, there may have been a halo effect leading one medical encounter with a good PPC to overall high ratings, or a recency effect could have resulted in patients who were treated most recently being more influential in physicians’ ratings. Furthermore, this study showed that there is variability in the ratings of patients clustered within each physician. This might be due to individual patient perspectives and preferences [50] as well as due to varied performance of physicians with different patients [51]. However, the current study is not able to explore this due to physicians’ generalised ratings of all encounters with their patients included in this study. Finally, five physicians completed a paper pencil questionnaire, whereas others completed an online questionnaire, which may have produced error variance but was not considered in this study.

### Conclusion and practice implications

PPC ratings only minimally reflect physicians’ communication. Furthermore, PCC ratings mostly reflect the interaction effects of rater perspective and communication dimension as well as the interaction effects of rater perspective and physician. This information has implications for settings where these types of rater variances are considered error sources. Consequently, rater training should be considered to reduce interrater disagreements [14]. In addition, measuring communication dimensions representing clear observational behavior requiring less rater inference would reveal more valid results. Additionally, researchers should provide detailed descriptions of the observed communication dimensions as well as pronounce which perspective was asked. This information will help decision makers and researchers draw conclusions about the quality of PPC. Finally, physicians tend to overestimate the extent of SDM they are practising. Hence, physicians should involve their patients more in SDM, even if they think they are already doing so sufficiently.

## Supporting information

S1 File Items of physician and patient questionnaire. (PDF)

S2 File Data set. (XLSX)
