# Supplementary material for: Variance components of ratings of physician-patient communication: A generalizability theory analysis
Source: PLoS One. 2021 Jun 10;16(6):e0252968. doi: 10.1371/journal.pone.0252968 (PMC8191893; doi:10.1371/journal.pone.0252968)
Supplement: S1 File — (PDF) [file pone.0252968.s001.pdf]

Patient version	Physician version (adapted)
SDM-Q [27-29]	
My doctor made clear that a decision needs to be made.	I made clear to my patients that a decision needs to be made.
My doctor wanted to know exactly how I want to be involved in making the decision.	I wanted to know exactly from my patients how they want to be involved in making the decision.
My doctor told me that there are different options for treating my medical condition.	I told my patients that there are different options for treating their medical condition.
My doctor precisely explained the advantages and disadvantages of the treatment options.	I precisely explained the advantages and disadvantages of the treatment options to my patients.
My doctor helped me understand all the information.	I helped my patients understand all the information.
My doctor asked me which treatment option I prefer.	I asked my patients which treatment option they prefer.
My doctor and I thoroughly weighed the different treatment options.	My patients and I thoroughly weighed the different treatment options.
My doctor and I selected a treatment option together.	My patients and I selected a treatment option together.
My doctor and I reached an agreement on how to proceed.	My patients and I reached an agreement on how to proceed.
Short version of the satisfaction scale of the P.A.INT questionnaire [30-32]	
I am satisfied with the result of the consultation.	I am satisfied with the results of the consultations.
I am satisfied with the process of the consultation.	I am satisfied with the processes of the consultations.
Effective and open communication scale of the KOVA questionnaire [33]	
Your physician listened carefully when you wanted to say something.	I listened carefully when my patients wanted to say something.
Your physician informed you at the end of treatment about the further treatment of your illness.	I informed my patients at the end of treatment about the further treatment of their illness.
Your physician informed you openly and directly of things concerning your illness that could be stressful (e.g. side effects of a treatment).	I informed my patients openly and directly of things concerning their illness that could be stressful (e.g. side effects of a treatment).
Your physician asked at the beginning of your treatment to explain all your symptoms.	I asked my patients at the beginning of their treatment to explain all your symptoms.
Your physician asked about all your symptoms.	I asked my patients about all their symptoms.
Your physician always told you everything about your illness, even if it is unpleasant.	I always told my patients everything about their illness, even if it is unpleasant.
Your physician asked you what you want to know about your treatment.	I asked my patients what they want to know about their treatment.
Your physician asked whether you experience pain during therapy/treatment.	I asked my patients whether they experience pain during therapy/treatment.
Your physician provided you the possibility to ask questions.	I provided my patients the possibility to ask questions.
Your physician explained to you exactly what your diagnosis means.	I explained to my patiens exactly what their diagnosis means.
